# Ubiquitin Modification Patterns of Clear Cell Renal Cell Carcinoma and the Ubiquitin Score to Aid Immunotherapy and Targeted Therapy

**DOI:** 10.3389/fcell.2021.659294

**Published:** 2021-05-13

**Authors:** Peng Zhou, Yuchao Lu, Yang Xun, Jinzhou Xu, Chenqian Liu, Qidong Xia, Junlin Lu, Shaogang Wang, Jia Hu

**Affiliations:** Department of Urology, Tongji Hospital, Tongji Medical College, Huazhong University of Science and Technology, Wuhan, China

**Keywords:** ubiquitin code, unsupervised consensus clustering, clear-cell renal-cell carcinoma, immune signature, immune checkpoint blockade, targeted therapy

## Abstract

Ubiquitin modification is the most common protein post-translational modification (PTM) process in organisms, and 1332 ubiquitin regulators have been identified in humans. Ubiquitin regulators, especially E3 ligases and deubiquitinases, are widely involved in immune processes. This study aims to explore the ubiquitin modification features of clear cell renal cell carcinoma (ccRCC) and to elucidate the role of such ubiquitin modifications in shaping anti-tumor immunity and individual benefits from immune checkpoint blockade (ICB). A comprehensive analysis was performed in the TCGA cohort (*n* = 530) and GEO cohort (*n* = 682). RNA sequencing data of 758 differentially expressed regulators, which was validated by the proteomics data, was used for k-means unsupervised consensus clustering and three ubiquitin patterns of ccRCC were identified. Then, we focused on the ubiquitin modification and tumor progression signatures, immune infiltration characteristics, and prognostic value. The three patterns with different ubiquitin modification signatures correspond to “immune desert phenotype,” “immune resistance phenotype,” and “immune-inflammatory phenotype,” respectively. To facilitate clinical application, we constructed a ubiquitin score to evaluate individual patients’ ubiquitination outcome, and it was demonstrated to be an independent risk factor for overall survival (OS) in multivariate Cox analysis. It was found that the high score group was correlated to higher immune cells infiltrating level and PD-1/PD-L1/CTLA-4 expression. More importantly, we found that the high score group was predicted to be sensitive to anti-PD-1 treatment, while the low-score group showed lower predicted IC50 values in treatment with Pazopanib and Axitinib. In summary, this study elucidated the potential link between ubiquitin modification and immune infiltration landscape of ccRCC for the first time and provided a new assessment protocol for the precise selection of treatment strategies for patients with advanced ccRCC.

## Introduction

Ubiquitin is a 76-amino acid small molecule protein that is highly conserved in sequence. The most common ubiquitination modification is sequentially catalyzed by ubiquitin-activating enzymes (E1s), ubiquitin-conjugating enzymes (E2s), and ubiquitin protein-ligases (E3s) (Kerscher et al., 2006). Ubiquitin itself can continue to bind ubiquitin molecules at multiple residues (i.e., K6, K11, K27, K29, K33, K48, K63, and Met1), thus forming complex structured ubiquitin chains on the substrates, known as the “ubiquitin code.” Besides, the ubiquitin-binding domain-containing protein (UBD) (Husnjak and Dikic, 2012), proteins containing ubiquitin-like domains (ULDs) (Upadhya and Hegde, 2003), and deubiquitinases (DUBs) (Nijman et al., 2005; Reyes-Turcu et al., 2009) act as “deciphers” of the “ubiquitin code” and negative regulators of this process. Accelerating evidence has shown that the dysregulation of the ubiquitin system plays a critical role in a variety of diseases, such as DNA repair damage, cellular autophagy, neurodegenerative pathologies, autoimmune diseases, and malignancies (Schwertman et al., 2016; Seeler and Dejean, 2017; Grumati and Dikic, 2018; Rape, 2018).

The expression of immune checkpoint molecules and the maturation of immune cells were regulated by the ubiquitin system. Meng et al. (2018) identified Lys48-linked polyubiquitination as the first post-translational modification (PTM) process of PD-1 and FBX038 as the mediator of the process. Lim et al. (2016) identified CSN5 as a DUB that inhibits the PD-L1 degradation. Blocking CSN5 with curcumin attenuated this inhibition and sensitized the cells to anti-CTLA4 treatment. Another study on triple-negative breast cancer (TNBC) identified β-TrCP as an E3 ligase participating in the poly-ubiquitination modification of PD-L1 (Li et al., 2016). Zhang et al. (2018) demonstrated that CDK4/6 degrades PD-L1 via Cullin3-SPOP E3 ligase in prostate cancer and the nonsense mutations of SPOP resulted in elevated PD-L1 expression level. Similar ubiquitin modification regulation was also found in the PTM process of LAG-3, CTLA4, and CD80/CD86 (Yao and Xu, 2020). Moreover, ubiquitin modifications also profoundly affected the maturation of immune cells and shaped the tumor microenvironment (TME) (Zhu et al., 2020). Alix et al. (2020) found that WWP2 blocked DC cell-induced T cell activation by targeting and degrading MHC-II expression in DC cells. A recent study showed that the deubiquitination enzyme Trabid can also affect DC cell-induced Th1 and Th17 cell differentiation by targeting the epigenetic regulation of IL-12/IL-23 (Jin et al., 2016). These studies indicated that ubiquitin modifications profoundly affected the fates of immune cells and the formation of an anti- or pro-tumorigenic microenvironment.

Renal cancer is a malignancy with a moderate mutation burden, but it dramatically responds to immune checkpoint blockade (ICB) therapy (Braun et al., 2020). Results from several clinical trials have shown that anti-PD-1/CTLA-4 combination therapy has a superior clinical effect over VEGFR-targeted therapy, marking a new era of immunotherapy for renal cell carcinoma (Grimm et al., 2020). Although there were abundant infiltrating T cells in clear cell renal cell carcinoma (ccRCC), the anti-tumor response was suppressed by Tregs and myeloid cells, resulting in inadequate durable benefit from ICB (Díaz-Montero et al., 2020). Indicated by the available evidence that ubiquitin system involving in the regulation of immune checkpoints (Hsu et al., 2018), an in-depth investigation of the ubiquitin patterns in ccRCC would further clarify the mechanism of immune resistance and help to identify reliable biomarkers of ICB responsiveness. The large number of ubiquitin regulators makes it difficult to depict the macroscopic immune landscape shaped by ubiquitination modifications of individual tumors using traditional research methods. Moreover, tumorigenesis is an interaction of multiple regulators in a highly coordinated manner, thus a more comprehensive and efficient analysis is needed to characterize the ubiquitin modifications in ccRCC. Based on this, we explored the ubiquitin patterns of ccRCC and comprehensively evaluated the underlying role in shaping immune maturation by analyzing the genomic information from a total of 1212 ccRCC samples. Herein, we identified three ubiquitin patterns in ccRCC, which correspond to three distinct immune phenotypes. Besides, we proposed a new ubiquitin score to evaluate samples’ ubiquitination modification outcomes and initially demonstrated its potentiality in predicting immunotherapy and targeted therapy responsiveness in this study.

## Materials and Methods

### ccRCC Datasets Collecting and Pre-processing

The datasets for this study were collected from the TCGA, GEO, and the Clinical Proteomic Tumor Analysis Consortium (CPTAC) databases. As discovery cohort, we downloaded the RNA sequencing data (read counts and FPKM values) and phenotype information of the TCGA-KIRC dataset^1^. Somatic mutation data of the TCGA dataset (*N* = 451) was downloaded from the cBioPortal website^2^. FPKM values were converted to TPM values for subsequent analysis, as it is identical to the microarray values (Wagner et al., 2012). To reduce noise, ubiquitin regulators with median absolute deviation values ≤ 0.5 were excluded. The testing cohort is composed of 5 Affymetrix GPL570 platform-based microarray datasets: GSE73731 (*N* = 265), GSE53757 (*N* = 144), GSE46699 (*N* = 130), GSE66272 (*N* = 54), and GSE36895 (*N* = 76). GPL10558 platform-based microarray datasets GSE65615 (*N* = 138) and GSE40435 (*N* = 202) were compiled as the external validating cohorts. We downloaded the original ‘‘CEL’’ files from the GEO database^3^, adjusted the background and quantile normalized the data sets using “RMA” algorithm of the “affy” package, and then removed the batch effect using the “ComBat” algorithm of the “sva” package to merge these datasets into one for validation (Johnson et al., 2007). For GSE29609 (*N* = 39), the expression matrix (normalized log10 values) and clinical information were directly downloaded and used to validate the prognostic value. Log ratio transformed proteomics data and the biospecimen features of ccRCC were download from the CPTAC website^4^ to validate the protein level of the ubiquitin regulators (Clark et al., 2019).

### Different Expressed Ubiquitin Regulators Analysis and Survival Analysis

Twenty-seven E1s, 109 E2s, 1153 E3s, 164 DUBs, 396 UBDs, and 183 ULDs were collected from the iUUCD 2.0 database (Gao et al., 2013), and there were 1332 regulators after duplication removal. DEG of ubiquitin regulators was performed in the discovery and testing datasets using “DESeq2” and “Limma” methods, respectively. DEGs of the discovery cohort were filtered at adjusted *p*-value < 0.01, and results of the testing cohort were screened at adjusted *p*-value < 0.05. Finally, 758 overlapped regulators were identified as the hub regulators in ccRCC. Significantly mutated regulators (*q* < 0.05) were inferred using the MutSigCV algorithm as described before (Lawrence et al., 2013). Prognostic values were assessed using univariate and multivariate-cox regression, and the survival differences were visualized using Kaplan-Meier curves.

### Identification of Ubiquitin Pattern and Molecular Characterization

Unsupervised consensus clustering of the 758 ubiquitin regulators was performed using the k-means algorithm, the cluster algorithm was set as “km,” and the similarity of samples was determined by “Euclidean” distance. This step was repeated 1000 times in the “ConsensusClusterPlus” package to ensure the stability of the classification (Wilkerson and Hayes, 2010). The 127 ubiquitin and proteasome-related biological processes were collected from the ‘‘c2.cp.kegg.v7.2.symbols’’ gene set (MSigDB database)^5^. “Gene Set Variation Analysis (GSVA)” method and “Limma” difference analysis were used for subsequent molecular characterization (Hanzelmann: 2013ga). Meanwhile, the “ClusterProfiler” package was used to annotate the function of each subgroup.

### Estimating the Immune Cell Infiltrating

Single sample gene set enrichment analysis (ssGSEA) is a method developed to estimate the relative abundance of immune cells based on the expression profile of a single sample. We obtained the gene set signatures of 28 immune cells (18 adaptive and 10 innate immune cell types) from the study of Charoentong et al. (2017), and the estimated score was calculated to represent the abundance of each cell type. CIBERSORT is an algorithm that deconvolves the expression matrix of bulk sequencing data based on the principle of linear support vector regression, and the sum of the percentages of each immune cell in the estimation result is 100% (Yoshihara et al., 2013; Newman et al., 2015). We used the “cibersort” package to analyze the discovery dataset, and samples with *p* < 0.05 in the results were included for comparison.

### Dimensional Reduction and Ubiquitin Score Generation

Here, we proposed to quantitatively assess the ubiquitin modification degree of ccRCC samples using the “ubiquitin score.” The ubiquitin score was derived as follows: Firstly, the Pearson correlation coefficients of 758 ubiquitin regulators with the identified ubiquitin patterns were calculated. Then the positively and negatively correlated genes were downscaled using the Boruta algorithm, respectively. Thus we obtained the signature genes A and signature genes B. Finally, the principal components analysis (PCA) was used to calculate the first principal components of signature genes A and B in each sample (Zhang X. et al., 2020). The ubiquitin scores of each sample were extracted as:

$$\mathrm{Ubiquitin}\mathrm{score}=\Sigma {\mathrm{PC1}}_{A}-\Sigma \mathrm{PC1}{}_{B}$$

### Predicting the Benefits of Ubiquitin Score for Immunotherapy and Targeted Therapy

The Tumor Immune Dysfunction and Exclusion (TIDE) is developed by Jiang et al. (2018) to predict the responsiveness to immunotherapy based on simulating tumor immune evasion mechanism. Due to the lack of open-access data of ccRCC cohorts accepting immunotherapy, we used the TIDE algorithm to preliminarily explore the responsiveness of the discovery cohort to ICB. Besides, we also used subclass mapping (Submap) to compare the similarity of gene expression profiles with 47 melanoma patients receiving anti-CTLA-4/PD-1 treatment to validate the results of TIDE prediction (Roh et al., 2017; Lu et al., 2019). Considering that VEGFR-targeted therapy remains the first-line treatment option for metastatic ccRCC (cc-mRCC), we explored the sensitivity of each subgroup to Sorafenib, Sunitinib, Pazopanib, and Axitinib. The tumor cell line genomic data and the corresponding IC50 of drug treatment from GDSC database^6^ were used as training dataset to estimate the IC50 values of tumor samples by ridge regression using the “pRRophetic” package, and the accuracy of the prediction results was assessed by 10-fold cross-validation (Geeleher et al., 2014).

### Statistical Analysis

All calculation and statistical analyses were performed in RStudio 3.6.3. Student’s *t*-test and Wilcoxon test were used for two-group comparison of normally or skewed distribution data, respectively. For multiple groups, Kruskal–Wallis test and one-way ANNOVA were used for parametric or non-parametric comparisons. Component differences in subgroups were compared by Fisher’s exact test. All statistical tests were two-sided, and *p* < 0.05 was considered statistically significant.

## Results

### Identification of the Differentially Expressed Ubiquitin Regulators and the Ubiquitin Patterns

**FIGURE 1:**
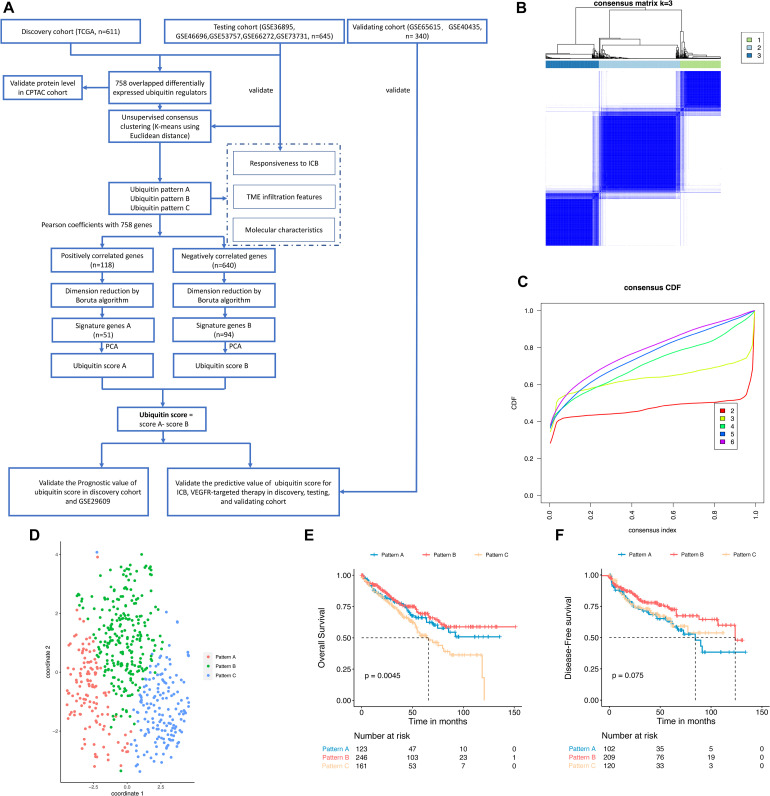
Identification of ubiquitin patterns using unsupervised consensus clustering and prognosis analysis in the discovery cohort. **(A)** The workflow of this study. **(B)** K-means clustering using 758 differentially expressed ubiquitin regulators. **(C)** CDF curve of the clustering result. **(D)** Validating the discrimination of *k* = 3 using t-SNE analysis. **(E,F)** Overall Survival (OS) and Disease-Free Survival (DFS) of the ubiquitin patterns in the TCGA-KIRC dataset. Statistical difference was compared using the log-rank test.

The analysis flowchart of this study was shown in Figure 1A. There were 947 differentially expressed ubiquitin regulators in the discovery dataset, and 1032 regulators differentially expressed in the testing cohort. 758 overlapped regulators shared by the two datasets were shown in Supplementary Figure 1A and detailed in Supplementary Table 1. To clarify that these regulators were similarly differentially expressed at the protein level, we checked the CPTAC dataset. In total, 562 regulators were involved in the proteomic data, 459 of which were statistically significant (*p* < 0.05), with a compliance rate of 81.68% (Supplementary Table 2). Subsequently, we explored the prognostic value of the 758 regulators for overall survival (OS) and disease-free survival (DFS) using univariate cox method (Supplementary Table 3).

To explore the ubiquitin patterns of ccRCC, unsupervised consensus clustering of the 758 regulators was performed. After comprehensive consideration of CDF curves and Delta area, we chose *k* = 3 as the number of subgroups (Figures 1B,C and Supplementary Figures 1B–F). In the discovery cohort, 123 patients were classified into Pattern A, 246 patients were classified into Pattern B, and 161 patients were classified into Pattern C. To verify the robustness of this classification, we used the t-SNE method for dimensional reduction and observed the discrimination of subgroups. As shown in Figure 1D, there was only individual cross-over, indicating good discrimination among subgroups. We also performed unsupervised consensus clustering in the testing cohort (Supplementary Figures 1G–N), and the results also showed three patterns of ubiquitin regulator expression in ccRCC samples. Regulators that were significantly higher expressed in each pattern (logFC > 0, adjusted *p*-value < 0.05) were identified as hub regulators of each pattern (Supplementary Figure 3A and Supplementary Table 4). In detail, there were 82 hub regulators for pattern A, 166 hub regulators for pattern B, and 264 hub regulators for pattern C. Besides, we found that these hub regulators were mainly composed of E3 ligases and UBD (Supplementary Table 5).

Then we compared the prognosis of the subgroups. The results showed that pattern B had a significant survival advantage with a median DFS time (123.7 months), while pattern A had the shortest median DFS time of 84.5 months (Figure 1F, log-rank test, *p* = 0.075). In pattern C we observed the shortest median OS of 65.7 months (log-rank test, *p* = 0.0045), while pattern A and B did not reach 50% median OS (Figure 1E). These results showed that the ubiquitin regulators in ccRCC exhibited three types of expression patterns, with each pattern possessing a different prognosis.

### Molecular Characteristics of the Distinct Ubiquitin Patterns

**FIGURE 2:**
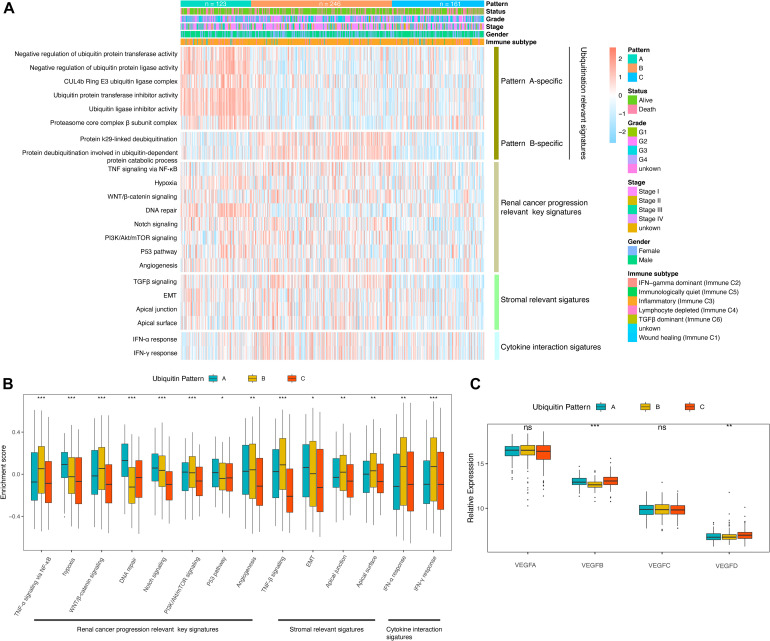
Molecular characteristics of the ubiquitin pattern. **(A)** Heatmap of the ubiquitin-relevant signature of the subclasses. **(B)** Boxplot of the GSVA enrichment score of ccRCC progression-relevant signatures. **(C)** Relative expression of VEGF family distinguished by ubiquitin patterns. The median values of the enrichment scores were compared using the Kruskal–Wallis test. Statistical significance levels were indicated with asterisks above the boxplot (ns, no statistical difference, * *p* < 0.05, ** *p* < 0.01, *** *p* < 0.001).

Considering that the classification is based on ubiquitin regulators, here we characterized the “ubiquitination code” signatures of each pattern. We calculated the enrichment scores of 127 ubiquitin and proteasome system-related biological processes using the GSVA algorithm, and ubiquitination relevant signatures of each pattern were defined as processes with higher enrichment scores in the limma analysis (log FC > 0.15, adjusted *p*-value < 0.05). The results showed the leading role of Culling-4b Ring E3 and proteasome complex β components and negative regulation of the ubiquitination process in pattern A. Pattern B is characterized by a deubiquitination process mediated by the K29 amino acid site. However, we did not find the ubiquitin-relevant signatures of pattern C under the criterion (Figure 2A).

**FIGURE 3:**
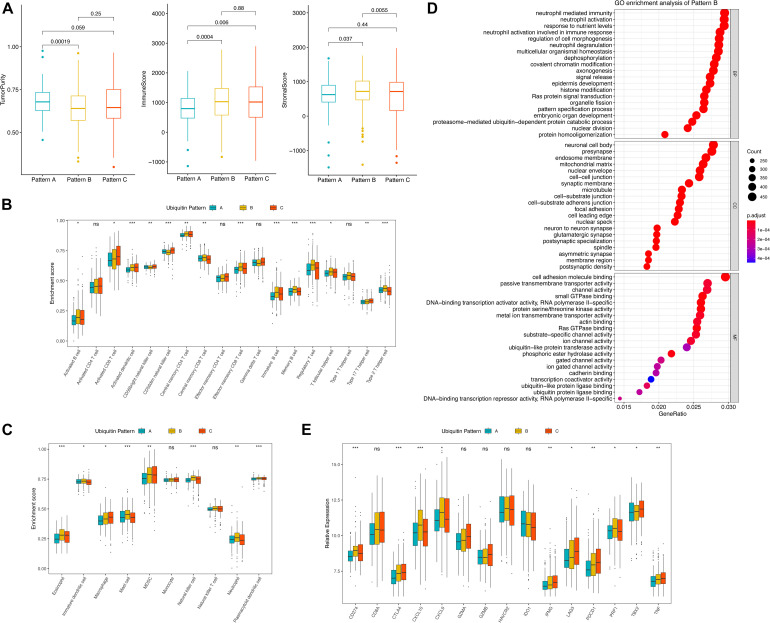
TME features of distinct ubiquitin patterns. **(A)** Tumor purity, immune score, and the stromal score of the subclasses generated by the “ESTIMATE” algorithm. The comparison was performed by Student’s *t*-test. **(B,C)** Estimated abundance of 18 adaptive and 10 innate immune cells using ssGSEA. Inter-subgroup comparison was performed by one-way ANOVA. **(D)** GO enrichment results of pattern B. The Top 20 of biological process (BP), cellular component (CC), and molecular function (MF) were displayed. **(E)** Association of ubiquitin patterns with immune checkpoint molecules. Kruskal–Wallis test, ns, no statistical difference, * *p* < 0.05, ** *p* < 0.01, *** *p* < 0.001.

Corresponding to the biological effects of distinct ubiquitin patterns, we further evaluated 14 renal cell cancer progression-relevant signatures. Patients in pattern A had higher DNA repair, p53, hypoxia, and EMT signaling pathway enrichment scores (Figures 2B,C), and activation of HIF-1 and Notch signaling were observed in GSEA analysis (Supplementary Figure 2A and Supplementary Table 6), suggesting greater tumor proliferation activity in pattern A, which explained the reason of shorter median DFS in pattern A (Figure 1F). Interestingly, key biological processes promoting kidney cancer progression such as angiogenesis, WNT, PI3K/Akt/mTOR signaling were more enriched in pattern B. On the other side, immune response-related signals such as pan-TNF and pan-IFN signaling were enriched in pattern B. GSVA analysis showed that pattern B exhibited both stromal activation and active immune response activity, suggesting a complex immune homeostatic mechanism in pattern B. We found that immune activation-related signals, such as antigen processing and presenting (APAP), NOD-like receptor (NLR), toll-like receptor (TLR), T-cell receptor (TCR), TNF were activated in pattern B (Supplementary Figures 2B,C). The GO enrichment results further characterized the leading role of neutrophil-mediated innate immunity in pattern B, At the same time, stroma-associated cellular components (CC) and molecular functions (MF) were also enriched in pattern B, which verified our speculation (Figure 3D).

### Tumor Microenvironment (TME) Infiltration Characteristics of the Distinct Expression Patterns

The imbalance of immune-related signatures among subtypes prompted further exploration of the immune infiltration profile. We firstly compared the tumor purity of the subtypes. By the ESTIMATE algorithm, we found a higher tumor purity of pattern A over pattern B and C, while no difference was found between pattern B and C. The immune scores of pattern B and pattern C were both higher than pattern A, indicating that the two groups had similar immune activation characteristics. In contrast, the stromal scores of pattern B were higher than pattern A and C, agreeing with the significant stromal activation of pattern B (Figure 3A). We then compare the proportion of immune cells among the three patterns (Supplementary Figure 2F). The results showed no statistical differences in the composition of immune cell types, suggesting that ubiquitin modification did not alter the overall TME infiltrating pattern (Fisher’s exact test, *p* = 0.924). Subsequently, we estimated the abundance of 18 adaptive immune cells and 10 innate immune cells in the samples using ssGSEA (Figures 3B,C). In general, pattern A showed a low abundance of almost all immune cell types in contrast to patterns B and C, and we termed it as “immune desert pattern.” Pattern B had more B cells, Treg, Th1, Th2, memory CD4+/CD8+ T cells, memory DC cells, and more neutrophils, NK cells, and other innate immune cells along with stromal activation, thus, corresponded to the “immune resistance phenotype.” Meanwhile, pattern C possessed more abundant Th17, activated CD4+/CD8+ T cells, DC cells, CD56+ NK cells, MDSC, and macrophages, corresponding to “immune-inflammatory phenotype.” However, patients in pattern C survived worst, which was inconsistent with the immune features of this subgroup (Figure 1E). One possible reason is that the anti-tumor response in pattern C was blocked by the simultaneous high expressed immune checkpoints. As we speculated, PD-1, CTLA4, GZMA, GZMB, IFNG, LAG3, TBX2, and TNF were higher expressed in pattern C (Figure 3E). The pair-wise comparison results revealed that these genes were significantly higher in group C compared with group A, while no significant difference existed when compared with group B except for TBX2 (Supplementary Table 7).

VHL mutation has been demonstrated to play an important role in ccRCC, but it is not clear whether it affects the immune landscape. MutSig results showed that the overall mutation rate of VHL was 50% in all samples, much higher than the other significantly mutated regulators (Supplementary Figure 3B). Therefore, we focused on exploring the potential role of VHL in the patterns we identified. There was no significant differences in VHL mutation rates among the three patterns (Supplementary Figure 3D, Fischer’s exact test, *p* = 0.448), but VHL expression levels were significantly lower in pattern A than in pattern B and C (Supplementary Figure 2C, Wilcoxon test, *p* = 1.1e-10, 9.6e-08, respectively). Furthermore, we found no significant difference in immune cell abundance between the mut/wild subtypes except for CD56bright NK cells. For PD-1, PD-L1, CTLA4, no statistical difference was found between the mut/wild VHL subtypes (Supplementary Figure 3H), which is in consistent with the findings of Hong et al. (2019).

### Correlation of the Ubiquitin Patterns With the Immunotherapy Benefits

**FIGURE 4:**
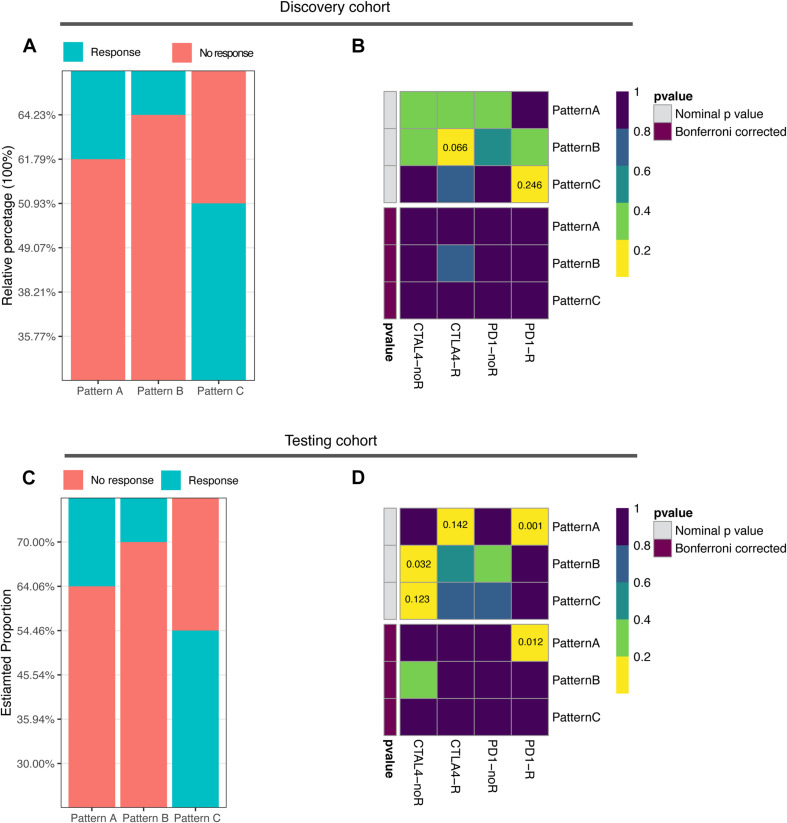
Correlation of ubiquitin patterns with immunotherapy benefit. **(A,C)** The predicted response rate in TIDE analysis of discovery and testing cohorts. Fisher’s exact test, *p* = 0.008, *p* < 0.0001, respectively. **(B,D)** The similarity of gene expression profiles between ubiquitin patterns and melanoma patients treated with ICB (*n* = 47). CTLA4-noR, patients no respond to anti-CTLA4 treatment, CTLA4-R, patients respond to anti-CTLA4 treatment, PD1-noR, patients no respond to anti-PD1 treatment, PD1-R, patients respond to anti-PD1 treatment.

The abundance of infiltrating immune cells and expression of immune checkpoint molecules in pattern C suggest the need to further explore the responsiveness of pattern C to ICB therapy. Based on the TIDE algorithm, we found significantly higher predicted response rates of pattern C (50.93%) in contrast to pattern A and B (38.21, 35.77%, respectively) (Figure 4A and Supplementary Table 10, Fisher exact test, *p* = 0.008). The testing cohort resulted similarly with a 54.46% predicted response rate of pattern A (Figure 4C and Supplementary Table 11, Fisher exact test, *p* < 0.0001). The expression profiles of each pattern were subsequently applied to Submap analysis. However, no definite similarity to ICB responders was found. None of the subgroups in the discovery cohort exhibited similarity to ICB responders, whereas pattern A in the testing cohort showed strong similarity to ICB responders (Figures 4B,D). This indicated the limitation and instability of population-based classification in predicting ICB treatment benefit.

### Ubiquitin Scores of Individual ccRCC Sample and the Prognostic Value

**FIGURE 5:**
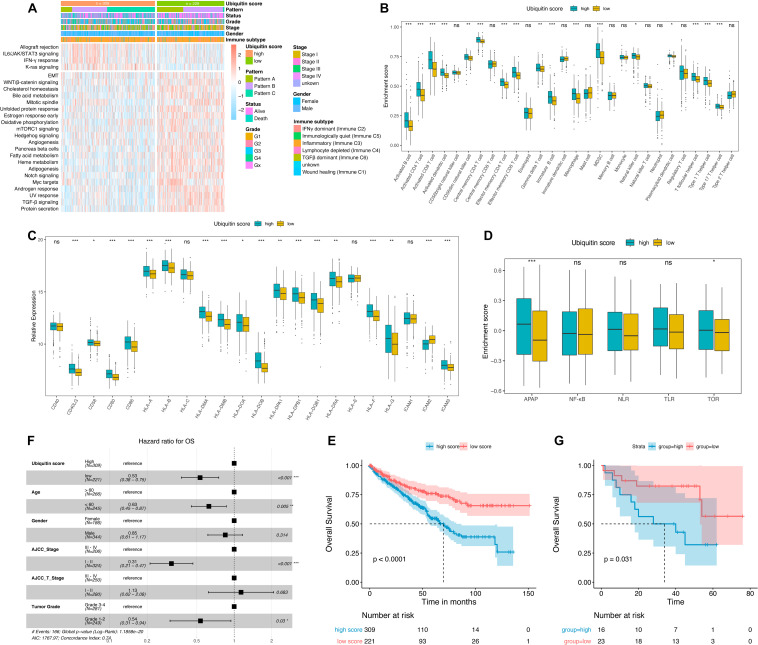
Characterization the ubiquitin score groups and prognosis analysis. **(A)** Hallmark pathways of ubiquitin score group determined by GSVA enrichment, | logFC| > 0.1. **(B)** Immune infiltration landscape, Wilcoxon test. **(C)** Relative expression of MHC molecules, co-stimulatory molecules, and adhesion factors, Wilcoxon test. **(D)** Enrichment scores of immune activation-related signatures, Wilcoxon test. APAP, antigen processing and presenting, NLR, NOD-like receptor signaling pathway, TLR, Toll-like receptor signaling pathway, TCR, T-cell receptor signaling pathway. **(E,G)** Kaplan-Meier curves of overall survival (OS) in TCGA **(E)** and GSE29609 **(G)** cohorts, Log-rank test. **(F)** Multivariate cox analysis adjusted by age, gender, tumor stage, T-stage, and tumor grade showed that ubiquitin score was an independent risk factor for OS. *p < 0.05, **p < 0.01, ***p < 0.001.

Previous studies demonstrated the close relationship between ubiquitin modification and anti-tumor immune activity. However, this patient population-based classification cannot accurately describe the ubiquitination outcome of the individual patients, which greatly limited its clinical application. Therefore, we continued to construct a ubiquitin score to quantify the ubiquitination outcome of single tumor sample. As the methods described, we downscaled the 758 regulators and obtained 51 and 94 genes that were positively and negatively associated with the ubiquitin patterns, which were termed as signature genes A and B, respectively (Supplementary Table 8). Supplementary Figure 4A displayed the expression landscape of 758 genes in each pattern. GO enrichment analysis showed that signature genes A were enriched in Culling3-Ring ligase, which was involved in protein poly-ubiquitination and phosphorylation modifications (Supplementary Figure 4B), while signature genes B were predominated in Culling-4 Ring E3 ligases, which was participating in proteasome-dependent protein degradation and deubiquitination process (Supplementary Figure 4C). The ubiquitin score was obtained by applying PCA performance to each signature gene (Supplementary Table 9). We compared the ubiquitin scores of the three patterns and found significant differences among the subgroups (Supplementary Figure 4D), with mode C having the highest ubiquitin score (median value of 5.079), mode A having the lowest score (median value of −3.931), and group B having an intermediate score (median value of −2.079). discovery cohort patients were classified into two groups using the best separation method, with 309 samples sorted into the high score group and 221 samples into the low score group. Prognosis analysis showed that the high score group had a significantly shorter median OS time (Figure 5E, *p* < 0.0001). To validate, the higher score group in the GSE29609 cohort also showed a significantly shorter median OS (Figure 5G, *p* = 0.031). Inclusion of the ubiquitin score along with the clinicopathological factors in multivariate analysis revealed that the ubiquitin score was an independent risk factor for OS (Figure 5F, HR = 1.47, *p* < 0.001). These results demonstrated the prognostic value of the ubiquitination score.

As shown in the heatmap (Figure 5A), IL6/JAK/STAT3, IFNγ, and K-ras signaling were upregulated in high score groups, while TGFβ signaling was downregulated (logFC > 0.1, adjusted *p*-value < 0.05). The majority of key signatures for renal cancer progression were enriched in the low score group, including EMT, WNT, mTORC1, Angiogenesis, Myc, and Hedgehog signaling. The high score group exhibited an advantage of activated CD4+/CD8+ T, MDSC, macrophages, and various types of DC cell infiltration (Figure 5B). Recent studies have shown that ubiquitinases (including E3 ubiquitinases and DUB) are key regulators of DC function (Jin et al., 2016). Activation of DC cells depends on the high expression of MHC molecules, co-stimulatory molecules, and adhesion factors (Qian and Cao, 2018). And we noted that high ubiquitin scores were accompanied by an overall elevation of MHC, adhesion molecules, and co-stimulatory molecules (Figure 5C). Subsequent comparison of immune activation-related pathways (including APAP, NFKB, NLR, TLR, and TCR) revealed a significant enhancement of APAP and TCR signaling in the high group (Figure 5D). These results demonstrated that ubiquitin modifications in ccRCC ultimately promote DC maturation and antigen presentation process.

### Correlation of the Ubiquitin Score With ICB Treatment Responsiveness and Targeted Therapy Sensitivity

**FIGURE 6:**
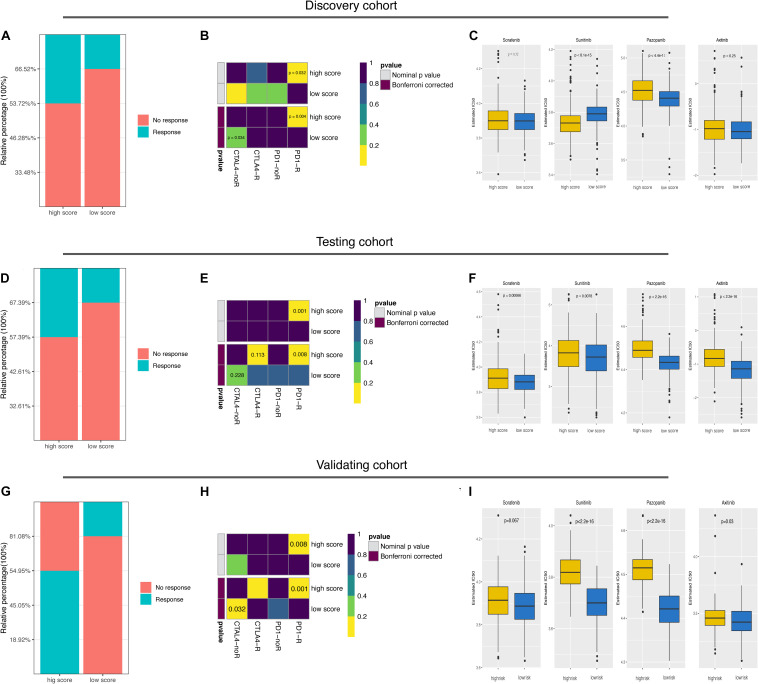
Predictive value of ubiquitin score for immunotherapy and targeted therapies. **(A,D,G)** The predicted response rate in TIDE analysis of the discovery, testing, and external validating datasets. Fisher exact test, *p* = 0.003, *p* = 0.034, *p* = 3.71e-08, respectively. **(B,E,H)** Subclass mapping results indicated that the high score group was more likely to respond to anti-PD-1 treatment (Bonferroni corrected *p*-value = 0.004, 0.008, 0.001, respectively). **(C,F,I)** Boxplots of the estimated IC50 values for Sorafenib, Sunitinib, Pazopanib, and Axitinib in the three cohorts, Wilcoxon test.

Finally, we explored the predictive value of the ubiquitin score to immunotherapy and targeted therapy. The TIDE results showed a higher predicted response rate in the high score group (46.28% vs. 33.48%, Figure 6A, *p* = 0.0032). In addition, we were delighted to see the consistent results with TIDE results in Submap analysis that the high score group was more likely to respond to anti-PD-1 treatment (Figure 6B, *p* = 0.032, 0.004, respectively). To validate, we generated the ubiquitin score for patients in the testing cohort. In the testing cohort, Pattern C had the highest ubiquitin score, while pattern A had the lowest ubiquitin score, with significant statistical differences in the pair-wise comparison (Supplementary Figure 4E). After dividing all patients into high/low score groups based on median ubiquitin score, the response rate was 42.61% in the high score group, while 32.61%in the low score group (Figure 6D, Fisher’s exact test, *p* = 0.034). The Submap analysis yielded positive results with high similarity in expression profiles between the high score group and the anti-PD-1 responders (Figure 6E, *p* = 0.001, 0.008, respectively). Because of the differential distribution of pattern A and B in high and low score groups, we performed subgroup analysis. For pattern A and B, there was no significant difference in responsive rates to ICB between the high and low subgroups (Fischer’s exact test, *p* = 1, *p* = 0.083, respectively). And there was no significant difference in response rate among the three patterns within the high score group (Fischer’s exact test, *p* = 0.151). For VHL subtypes, no significant difference in the ubiquitin scores between the mut/wild subtypes was found (Supplementary Figure 3E, Wilcoxon test, *p* = 0.12). Accordingly, TIDE results showed no significant difference in responsiveness to ICB between the VHL subtypes (Supplementary Figure 3F, Fischer’s exact test, *p* = 0.146).

Considering that targeted therapy remains the first-line treatment option for advanced renal cell cancer, we evaluated the sensitivity to Sorafenib, Sunitinib, Pazopanib, and Axitinib in both groups. Prediction models were trained on the GDSC cell line dataset by ridge regression and validated by 10-fold crossover to make the prediction results stable. IC50 values were estimated for each sample and the differences were compared. In the discovery cohort, we found that the high score group was more likely to be sensitive to Sunitinib (*p* < 8.1e-15), while the low score group more sensitive to Pazopanib and Axitinib (Figure 6C, *p* < 4.4e-11, *p* = 0.25, respectively). In the testing cohort, the low score group showed a lower predicted IC50 value in treating with the four candidate drugs (Figure 6F). Since mTOR signaling, VEGFR family, PDGFR family, and KIT expressed higher in the low score group (Supplementary Figure 3G), the above prediction results were reasonable.

Lastly, these findings were all validated in an external independent cohort. The results of the TIDE and Submap analysis again demonstrated that the high ubiquitin score group may respond to ICB treatment, while the low score group is more sensitive to Sunitinib, Pazopanib, and Axitinib (Figures 6G–I and Supplementary Table 12). All in all, these results firmly proposed that ubiquitin scores to be used to predict patient benefits from ICB and targeted therapy.

## Discussion

The role of ubiquitin regulators in ccRCC has been of interest to researchers since the E3 ubiquitin ligase pVHL deficiency was identified as an essential feature of ccRCC (Gossage et al., 2015). Recently, Guo et al. (2012) identified 12 novel high-frequency mutated genes that were enriched in the ubiquitin-mediated protein hydrolysis pathway by whole exon sequencing (WES) assay, and these genes were closely associated with overexpression of HIF factors. In addition, ubiquitin factors involved in key signaling of renal cell cancer, such as p53, PI3K/Akt, and Angiogenesis, are being increasingly identified (Ma et al., 2015; Guo et al., 2019; Hao et al., 2019; Yu et al., 2019; Zhang E. et al., 2020). However, there was a small number of studies investigating the role of ubiquitin regulators on the immune system of ccRCC. To our knowledge, this is the first time to comprehensively assess the ubiquitin modification pattern of renal cell carcinoma and to characterize their biological outcomes, especially the ubiquitin regulator-mediated immune features using bioinformatics method. The newly published proteomics data provide important supporting evidence for our study (Clark et al., 2019). Due to the limitations of detection technology and rapid protein degradation, proteomic data usually have a large disparity with the transcriptomic data. In the CPTAC ccRCC dataset, only 9964 proteins were detected in total, which is half amount of the coding genes. However, 562 of the 758 ubiquitin regulators used in our study were detected, with a detection rate of 74.14% and a compliance rate of 81.68%. Based on this, we suggest that it is meaningful to use RNA seq data for subsequent analysis.

In contrast to the conventional perception of immunology, highly infiltrative macrophages, Treg, and CD8+ T cells in ccRCC tend to be associated with worse oncologic outcomes (OS and PFS) (Bruni et al., 2020). The study by Braun et al. (2020) further showed that although about 73% of advanced ccRCC was infiltrated by CD8+ T cells, this high infiltration status was not associated with anti-PD-1 treatment benefit. It was demonstrated that the presence of pro-angiogenic, pro-inflammatory TME in ccRCC induced upregulation of multiple immune checkpoint expression on CD8+ toxic T cells, which present an “immune depleted phenotype” (Nakano et al., 2001; Giraldo et al., 2015; Granier et al., 2017). On the contrary, CD8+ T cells were more active in patients with a lower level of vascular factors, and these patients had a better oncology outcome (Giraldo et al., 2015). This suggests that renal cell carcinoma progression-relevant signatures were negatively associated with immune activation signatures. In our study, both the ubiquitin pattern C and the high score group had an immune infiltration advantage but worse OS and PFS, which is in line with the previously described phenomenon. In the comparison of the VEGF superfamily, pattern C was found to have a significantly higher expression of VEGFB and VEGFD (Figure 2C). Thus, high level of vascular factors and immune checkpoints blocked its anti-tumor immune response, ultimately lead to the worst prognosis of pattern C. Interestingly, however, these patients with the worst prognosis were most likely to benefit from ICB. The TIDE results showed a predicted response rate of 50.93% in pattern C, which was higher than 35.77% in pattern B and 38.21% in pattern A. Among the three patterns, pattern A lacked infiltrating immune cells, immune cells of pattern B were trapped in the stroma and cannot actually reach the tumor cells, and only the immune cells in pattern C infiltrated into the tumor nest, therefore anti-tumor immunity was restored the best when drugs unlocked the immune checkpoint.

In advantage of the “Boruta” algorithm and PCA analysis, we generated the ubiquitin score of single patients and demonstrated its prognostic value. Analysis of the signature genes revealed the prominent role of Culling-Ring ubiquitin ligase (E3), ubiquitin-protein transferase (E2), and ubiquitin-like protein protease (ULD) in the ubiquitin system of ccRCC (Supplementary Figures 3B,C). Besides, we found that signature gene A was enriched to protein phosphorylation modification process in ccRCC, and the oxidative phosphorylation pathway was down-regulated in the high ubiquitin score group, suggesting that the phosphorylation process may be involved in the ubiquitin modification process of ccRCC (Supplementary Figures 3B and Figure 5A). Small ubiquitin-like modifiers (SUMOs), including SUMO1/SUMO2/SUMO3/SUMO4/WDR48, are intra-nuclear PTM regulators well-studied in recent years. The results of a recent proteomics study showed that the intra-nuclear modification sites of SUMOs are mainly determined by pre-existing phosphorylation events, and these co-modification processes are regulated by cell cycle protein-dependent kinases (Hendriks et al., 2017). In the difference analysis of the GSVA enrichment scores, we found that signals related to immune shaping and cytokine responses, such as IL6/JAK/STAT3 and IFN-γ signaling, were more enriched in the high score group while signaling related to proliferation and epithelial-mesenchymal transition were downregulated. Notably, TGFβ signaling was negatively correlated to ubiquitination signaling (Figure 5A), and Fukasawa et al. (2010) showed that the ubiquitination degradation process of TGFβ-RII mediated by Smurf2 was significantly enhanced in renal cell cancer, which might be the reason for the TGFβ signaling attenuation in ccRCC.

In the latest edition of EAU guidelines, the anti-PD-1/CTLA4 combination treatment is recommended as the first-line treatment option for high-risk cc-mRCC patients (Ljungberg et al., 2020). In Phase 3 clinical trial of CheckMate-214 (NCT02231749), anti-PD-1 antibody Nivolumab combined with anti-CTLA-4 antibody Ipilimumab resulted in an overall response rate (ORR) of 41.6% (OS in 18 months was 75%) in the treatment of advanced renal cancer (Motzer et al., 2018). Despite these advances, reliable biomarkers of ICB therapeutic efficacy remain for further discussion. The instability of a single biomarker to predict benefit from immunotherapy strategies is now recognized. In our study, almost all patients in pattern C and part of patients from pattern B and A with high ubiquitin scores were categorized into high score group, which had both an anti-tumor immune infiltration advantage and high expression of immune checkpoints (Figures 5B–D and Supplementary Figure 3F). Therefore, it is reasonable that the high score group has a higher response rate to ICB treatment. The predicted response rates of 46.28, 42.61, and 36.96% in the three cohorts were close to the result of CheckMate-214, which strengthened our confidence in the predictive value of ubiquitin score. Meanwhile, we observed consistent trends of PD-1/PD-L1/CTLA-4 expression in the high score group, so we suggest it more appropriate to be used to assess the patients’ benefit from the anti-CTLA-4/PD-1 combination therapeutic strategy. In regard to this, further validation of the veracity should be performed in a ccRCC dataset receiving immunotherapy. The latest guidelines raise the recommendation grade (1b) of Pembrolizumab and Axitinib for the first-line treatment option for low- and intermediate-risk cc-mRCC patients, while Sunitinib (1b) and Cabozantinib (2a) were recommended as an alternative for patients who cannot tolerate or receive ICB treatment, and Pazopanib (1b) only recommended for intermediate-risk patients (Ljungberg et al., 2020). In particular, the combination of Pembrolizumab and Axitinib, approved for m-ccRCC treatment in 2019, may bring an exciting shift to the therapeutic field (Rini et al., 2019). For Sunitinib, the discovery cohort showed an opposite result with testing and validation cohorts. Analysis of the target molecules revealed that the predicted IC50 values in the TCGA dataset contradicted the expression level of VEGFR and PDGFR (Supplementary Figure 3G). Considering the fact that tumor proliferation and mTOR signaling were more active in the low score group, it was more reasonable than the high score group to be more sensitive to Sunitinib. Limited by the types of candidate drugs currently available in the algorithm, we could not estimate the IC50 values of Pembrolizumab and Cabozantinib in this study. In a recent study including 91 patients with cc-mRCC (treated with Nivolumab or Sunitinib), neither transcriptome nor exome sequencing data showed a correlation between VHL and clinical benefit, and our predicted results were consistent with that fact (Dizman et al., 2020).

## Conclusion

In conclusion, we identified three ubiquitin patterns in ccRCC with different oncological outcomes, which had distinctly different immune characteristics and prognostic outcomes. In clinical application, the “ubiquitin score” could be used to predict patients’ responsiveness to immunotherapy (high score group) and sensitivity to Pazopanib and Axitinib (low score group). Our study illustrated the key role of ubiquitin regulators in the TME of ccRCC and immunotherapy outcome, and provided a new reference for the management strategies of advanced ccRCC.

## Data Availability Statement

The datasets presented in this study can be found in online repositories. The names of the repository/repositories and accession number(s) can be found below: https://www.ncbi.nlm.nih.gov/geo/, GSE53757; https://www.ncbi.nlm.nih.gov/geo/, GSE46699; https://www.ncbi.nlm.nih.gov/geo/, GSE66272; https://www.ncbi.nlm.nih.gov/geo/, GSE36895; https://www.ncbi.nlm.nih.gov/geo/, GSE73731; https://www.ncbi.nlm.nih.gov/geo/, GSE29609; https://portal.gdc.cancer.gov/repository, TCGA-KIRC; https://www.ncbi.nlm.nih.gov/geo/, GSE65615; and https://www.ncbi.nlm.nih.gov/geo/, GSE40435.

## Author Contributions

SW and JH proposed and designed the framework of this study. JX, QX, and CL completed the collation and pre-processing of the raw data required for subsequent analysis. PZ performed a detailed analysis of the data, drafted this manuscript. YX, JL, and YL reviewed and critically revised the manuscript. The manuscript was confirmed by all authors before submitted for peer review.

## Conflict of Interest

The authors declare that the research was conducted in the absence of any commercial or financial relationships that could be construed as a potential conflict of interest.

## Footnotes

1 https://portal.gdc.cancer.gov/repository

2 http://www.cbioportal.org/study/summary?id=kirc_tcga

3 http://www.ncbi.nlm.nih.gov/geo/

4 https://cptac-data-portal.georgetown.edu/study-summary/S044

5 https://www.gsea-msigdb.org/gsea/msigdb/collections.jsp#C5

6 https://www.cancerrxgene.org/

## Supplementary Material

Supplementary Figure 1 Unsupervised consensus clustering in GEO-KIRC datasets. **(A)** The 758 overlapped differentially expressed ubiquitin regulators in the Venn diagram. **(B–F)** Consensus clustering results when *k* = 2, 4, 5, 6 in the discovery cohort and delta area plot. **(G–M)** Consensus clustering result when *k* = 2, 3, 4, 5, 6 in the testing cohort. **(N)** t-SNE result of the testing cohorts.

Supplementary Figure 2 GSEA of ubiquitin patterns. **(A–E)** The enrichment results by GSEA for patterns A, B, and C. **(F)** Comparison of immune infiltration difference among subgroups by CIBERSORT deconvolution, Fisher exact test, *p* = 0.924.

Supplementary Figure 3 Correlation of VHL mut/wild subtypes with ubiquitin patterns and immunotherapy responsiveness. **(A)** Heatmap of the distinct patterns’ hub regulators. 82 regulators for pattern A, 166 regulators for pattern B, and 264 regulators for pattern C. **(B)** Significantly mutated ubiquitin regulators in ccRCC inferred by MutSigCV method. The oncoplot showed that VHL was the most frequently mutated ubiquitin regulators with a 50% mutation rate. **(C)** VHL expression and **(D)** mutation status of the three patterns. There was no significant difference in the proportion of VHL mutant phenotype among the three patterns, *p* = 0.448, Fischer’s exact test. **(E)** No difference of ubiquitin score between the VHL mut/wild subtypes, Wilcoxon test, *p* = 0.12. **(F)** No difference of immunotherapy response rate between the VHL mut/wild subtypes was found by TIDE method, *p* = 0.146, Fischer’s exact test. **(G)** Immune cell abundance between the VHL mut/wild subtypes, Wilcoxon test. **(H)** PD-L1(CD274), CTLA4, PD-1(PDCD1) expression level between the VHL mut/wild subtype, Wilcoxon test.

Supplementary Figure 4 Generation of the ubiquitin score. **(A)** heatmap of the positive and negatively correlated regulators in three ubiquitin patterns. **(B,C)** GO enrichment of signature gene A and B. **(D,E)** ubiquitin scores of distinct ubiquitin pattern in the discovery **(D)** and testing **(E)** cohorts, pair-wise comparison using Wilcoxon test. **(F,G)** Drug targets of immunotherapy **(F)** and VEGFR-targeted therapy **(G)** expression level, Wilcoxon test.

## Abbreviations

PTM: post-translational modification
ccRCC: clear cell renal cell carcinoma
ICB: immune checkpoint blockade
OS: overall survival
E1s: ubiquitin-activating enzymes
E2s: ubiquitin-conjugating enzymes
E3s: ubiquitin protein-ligases
UBD: ubiquitin-binding domain-containing protein
ULDs: ubiquitin-like domains
TNBC: triple-negative breast cancer
DC: dendritic cell
PCA: principal components analysis
DFS: disease-free survival
CC: cellular components
MF: molecular functions
TME: tumor microenvironment
WES: whole exon sequencing
SUMOs: Small ubiquitin-like modifiers
ORR: overall response rate
TMB: tumor mutational load
APAP: antigen processing and presenting
NLR: NOD-like receptor signaling pathway
TLR: toll-like receptor signaling pathway
TCR: T-cell receptor signaling pathway
CPTAC: Clinical Proteomic Tumor Analysis Consortium
GSVA: Gene Set Variation Analysis
ssGSEA: single sample gene set enrichment analysis.
