# Supplementary figures and images for: Ubiquitin Modification Patterns of Clear Cell Renal Cell Carcinoma and the Ubiquitin Score to Aid Immunotherapy and Targeted Therapy
Source: Front Cell Dev Biol. 2021 May 13;9:659294. doi: 10.3389/fcell.2021.659294 (PMC8158301; doi:10.3389/fcell.2021.659294)

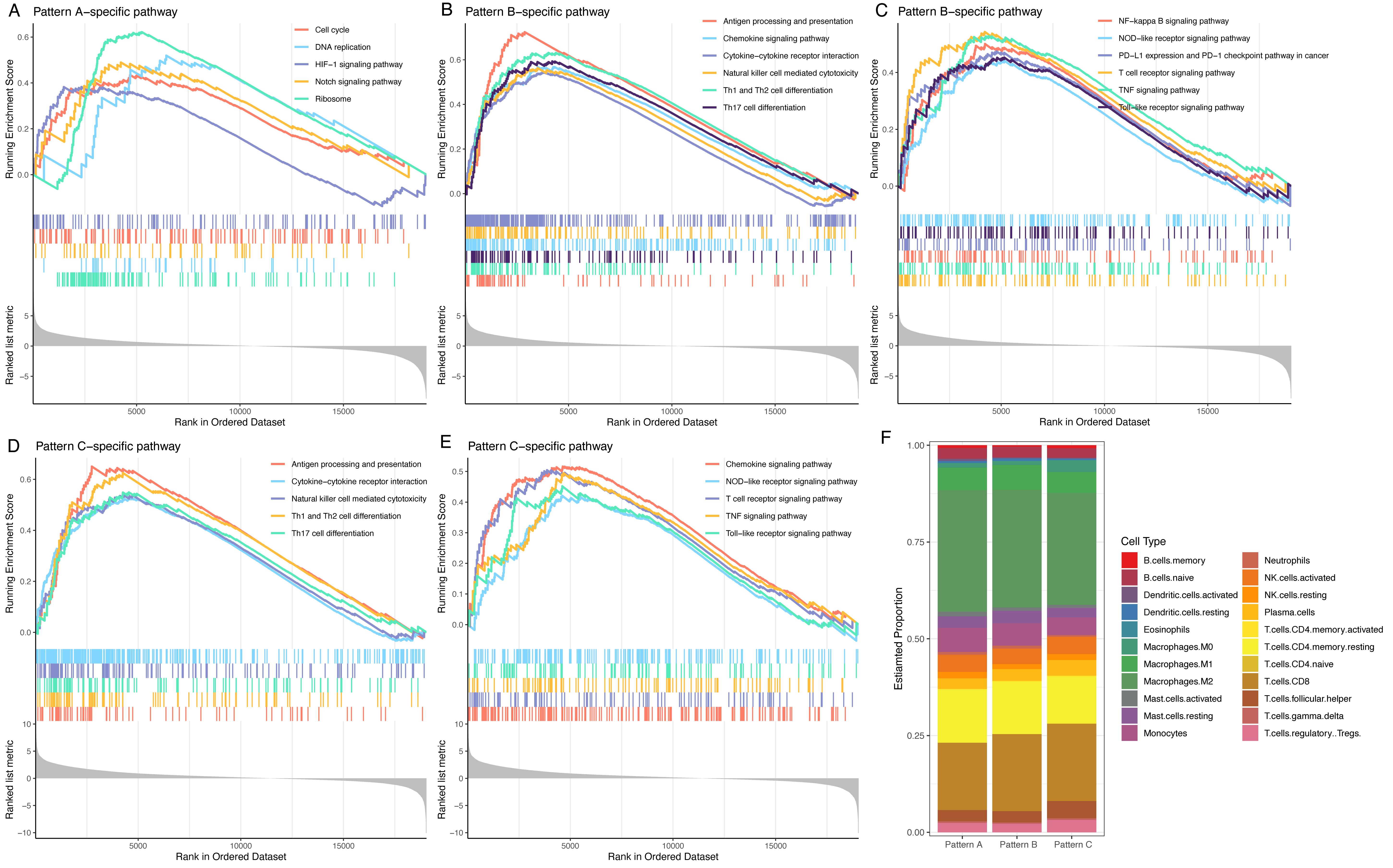

Supplement: Supplementary Figure 2 — GSEA of ubiquitin patterns. (A–E) The enrichment results by GSEA for patterns A, B, and C. (F) Comparison of immune infiltration difference among subgroups by CIBERSORT deconvolution, Fisher exact test, p = 0.924. [file Image_2.tif]

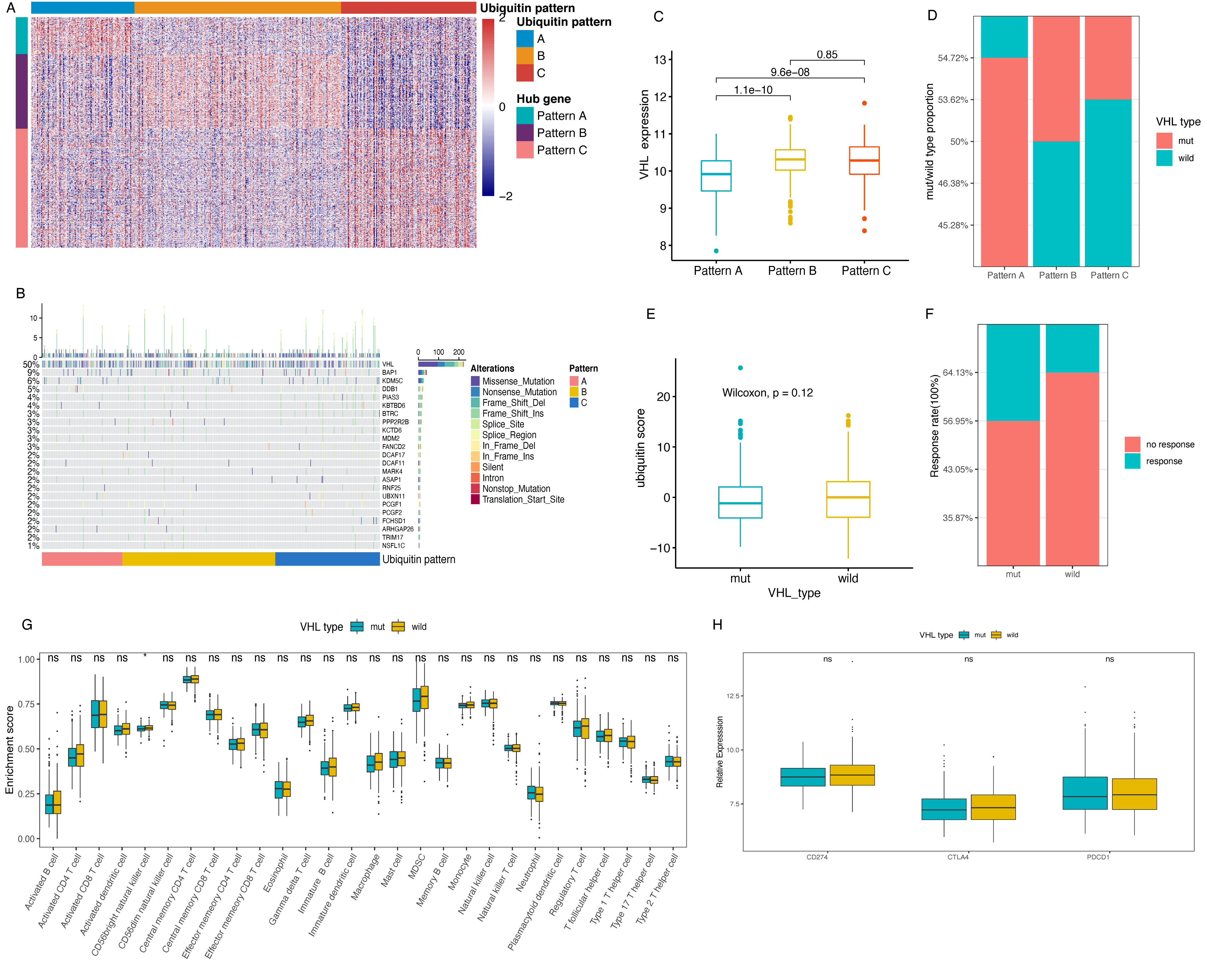

Supplement: Supplementary Figure 3 — Correlation of VHL mut/wild subtypes with ubiquitin patterns and immunotherapy responsiveness. (A) Heatmap of the distinct patterns’ hub regulators. 82 regulators for pattern A, 166 regulators for pattern B, and 264 regulators for pattern C. (B) Significantly mutated ubiquitin regulators in ccRCC inferred by MutSigCV method. The oncoplot showed that VHL was the most frequently mutated ubiquitin regulators with a 50% mutation rate. (C) VHL expression and (D) mutation status of the three patterns. There was no significant difference in the proportion of VHL mutant phenotype among the three patterns, p = 0.448, Fischer’s exact test. (E) No difference of ubiquitin score between the VHL mut/wild subtypes, Wilcoxon test, p = 0.12. (F) No difference of immunotherapy response rate between the VHL mut/wild subtypes was found by TIDE method, p = 0.146, Fischer’s exact test. (G) Immune cell abundance between the VHL mut/wild subtypes, Wilcoxon test. (H) PD-L1(CD274), CTLA4, PD-1(PDCD1) expression level between the VHL mut/wild subtype, Wilcoxon test. [file Image_3.tif]

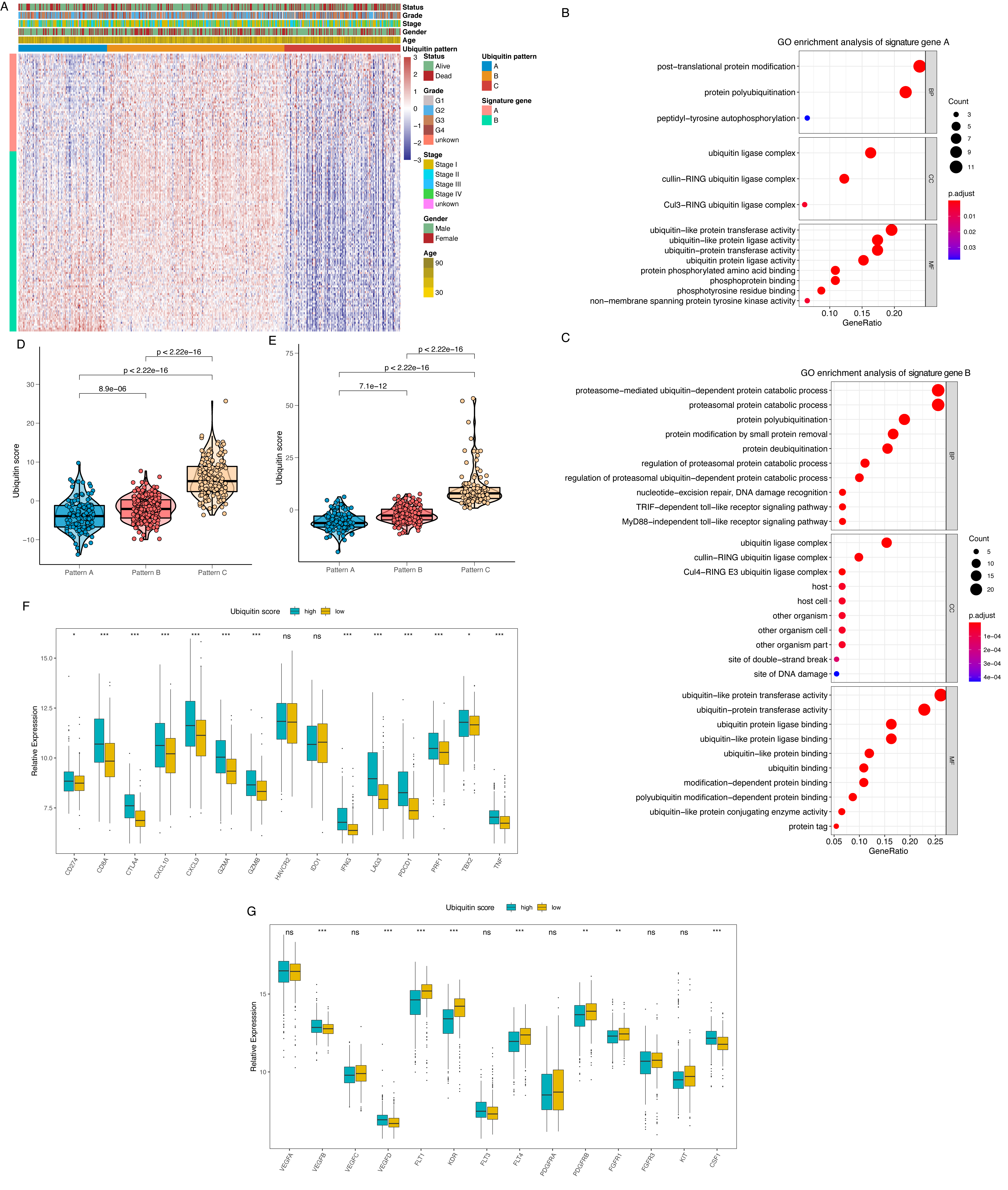

Supplement: Supplementary Figure 4 — Generation of the ubiquitin score. (A) heatmap of the positive and negatively correlated regulators in three ubiquitin patterns. (B,C) GO enrichment of signature gene A and B. (D,E) ubiquitin scores of distinct ubiquitin pattern in the discovery (D) and testing (E) cohorts, pair-wise comparison using Wilcoxon test. (F,G) Drug targets of immunotherapy (F) and VEGFR-targeted therapy (G) expression level, Wilcoxon test. [file Image_4.tif]
